# A single residue in domain II of envelope protein of yellow fever virus is critical for neutralization sensitivity

**DOI:** 10.1128/jvi.01770-24

**Published:** 2025-02-28

**Authors:** Ya-Nan Lou, Meng-Xu Sun, Kai Li, Xiao-Chuan Xiong, Chao Zhou, Tian-Shu Cao, Xiao-Feng Li, Cheng-Feng Qin

**Affiliations:** 1 State Key Laboratory of Pathogen and Biosecurity, Academy of Military Medical Sciences 602528 , Beijing, China; 2 Research Unit of Discovery and Tracing of Natural Focus Diseases, Chinese Academy of Medical Sciences 12501 , Beijing, China; University of North Carolina at Chapel Hill, Chapel Hill, North Carolina, USA

**Keywords:** yellow fever, vaccination, neurovirulence, neutralization escape

## Abstract

The live-attenuated yellow fever (YF) vaccine strain 17D (referred to as YF-17D) is the only commercially available vaccine against YF. Recent yellow fever virus (YFV) isolates of South American genotype I (SAI) showed reduced sensitivity to sera from individuals vaccinated with YF-17D. However, the underlying mechanisms remain inadequately elucidated. Here we conducted a screening of a panel of amino acid changes prevalent in domain II of the envelope protein (EDII) of SAI YFV isolates. Each mutation was introduced into the infectious clone of YF-17D utilizing standard reverse genetics. Subsequent neutralization assays demonstrated that the A83E mutant was more sensitive to sera from YF-17D-vaccinated mice and individuals than YF-17D did. Additionally, the A83E mutant displayed neurovirulence and viscerotropic characteristics comparable to those of the parental YF-17D in murine models. Notably, administration of a single dose of the A83E mutant resulted in an enhanced neutralization response against this variant compared to YF-17D and provided complete protection against lethal YFV challenge in mice. Collectively, these findings not only identify a crucial amino acid residue that influences the neutralization sensitivity of YFV but also provide a promising candidate for the development of an updated YF vaccine.

**IMPORTANCE:**

The YF-17D vaccine has been used to prevent YF disease. However, recent strains belonging to the SAI displayed reduced sensitivity to the antibodies produced by vaccination, raising concerns about potential future outbreaks. To identify potential amino acid residues responsible for the decreased neutralizing activity of YF-17D-vaccinated sera, we conducted a screening and generated recombinant viruses with amino acid changes specific to the SAI in EDII using the YF-17D genome as a genetic backbone. We found the A83E mutation played a key role in reducing neutralizing sensitivity to YF-17D-vaccinated mouse sera. Importantly, the A83E mutant maintained a comparable attenuation phenotype to YF-17D but elicited enhanced neutralization activity and conferred protection in mice. Together, we identify a key amino acid residue responsible for the neutralization escape of SAI YFV isolates. We propose that this substitution could act as a target for developing an updated YF-17D vaccine.

## INTRODUCTION

Yellow fever virus (YFV), the causative agent of yellow fever (YF) disease, belongs to the genus *Orthoflavivirus* and family *Flaviviridae*, which also includes other important human pathogens such as dengue virus (DENV), Japanese encephalitis virus, West Nile virus (WNV), and Zika virus. YF is endemic to tropical and subtropical areas of Africa and Central and South America. According to the World Health Organization (WHO), a total of 47 countries (34 countries in Africa and 13 countries in the Americas) are considered to be at highest risk of YF (1). Phylogenetic studies have classified YFV diversity into seven genotypes. The five African lineages include two West African genotypes, a single Central/South African genotype, and two East African genotypes. Furthermore, there are two South American genotypes (South American genotype I [SAI] and South American genotype II) which were derived from an ancestral West African lineage (2–6). YFV is transmitted to humans through the bite of infected *Aedes* mosquitoes in Africa and *Hemagogus* mosquitoes in South America (7, 8). Clinical manifestations of YF in humans can range from inapparent or mild disease to potentially fatal hemorrhagic disease, including high fever, vomiting, jaundice, dark urine, shock, and organ failure (8). YFV continues to cause periodic, large outbreaks in tropical regions of Africa and South America, with an estimated case fatality rate of 20%–50% in patients with severe disease (9). Notably, increasing exchanges between Africa and Asia have resulted in imported YFV cases in China in 2016 (10). Despite no onward transmission in China, over 2 billion people in Asia live in areas inhabited by *Aedes aegypti*, underscoring the potential emergence of a new epidemic disease threat in Asia (11).

Immunization is considered to be the most important and effective measure for preventing YF. Currently, the licensed YF vaccine is based on the live-attenuated 17D virus (referred to as YF-17D), which was derived from the virulent African strain Asibi and was further passaged to yield YFV vaccines prequalified and stockpiled by the WHO (12). Since its introduction in 1939, YF-17D has been administered to over 850 million individuals globally with a high efficacy and acceptable safety profile (13). Despite the availability of this safe vaccine, massive YF outbreaks occurred recently in both Africa (Angola in 2015–2016 and Democratic Republic of Congo in 2016) and South America (Brazil in 2016–2019) (14). Multiple factors are believed to contribute to the reemergence of YFV in South America and elsewhere, including urbanization, large population movements, climate change, increasing exposure of workers to infected mosquitoes in jungles and forests, and inadequate vaccination coverage (1). Notably, genomic analyses of YFV responsible for recent outbreaks revealed unique mutations leading to nine amino acid substitutions in the deduced polyprotein (15). The genetic divergence between YFV genotypes is believed to play a significant role in the differing epidemiological patterns of the disease (16). Furthermore, some studies in cell cultures and/or animal models point to differences in replicative ability and virulence among YFV strains from different genotypes (17–20). Recently, Haslwanter et al. showed that sera from YF-17D vaccinees had reduced neutralization sensitivity to the SA isolates, including the emergent strain ES-504 (21). These observations highlight the necessity of identifying the critical residues of YFV that contribute to the reduced neutralizing capability against the SA isolates.

Like other flaviviruses, YFV is an enveloped positive-sense single-stranded RNA virus that encodes a single open reading frame flanked by 5′ and 3′ untranslated regions. The genome of YFV is approximately 11 kb in length and is translated as a single polyprotein. This polyprotein undergoes post-translational modifications and cleavage by both host and viral proteases, resulting in three structural proteins (capsid, premembrane, and envelope [E]) and seven non-structural proteins (NS1, NS2A, NS2B, NS3, NS4A, NS4B, and NS5) (22). The E protein, the primary surface-exposed protein in mature particles, is responsible for virus binding, entry, and fusion in host cells (23). It consists of three domains (domain I of the envelope protein [EDI] to domain III of the envelope protein III [EDIII]) connected to the viral membrane by a helical stem and two transmembrane domains. EDI is the central domain, and EDII contains various immunodominant epitopes, including the fusion loop (FL) responsible for viral and host membrane fusion (24). EDIII contains the putative cellular attachment domain with an immunoglobulin-like structure (25). Generally, type-specific neutralizing antibodies (nAbs) against individual flaviviruses primarily localize to EDIII, and flavivirus cross nAbs target the EDI/EDII hinge regions and EDII (26–33). In the case of YFV, most neutralizing antibodies induced by YF-17D vaccination are YFV specific (34). Mapping of neutralizing epitopes on the YFV-E protein using mouse monoclonal antibodies has previously identified critical amino acid residues in EDII, EDIII, and EDI (23). In individuals vaccinated with YF-17D, the immune response predominantly targets EDII, with only a minor fraction targeting EDIII (35, 36). Based on human nAb analysis, Haslwanter et al. have identified two sites within EDII of the South American isolates that contribute to the reduced neutralization sensitivity (21). Further characterization with the reporter viral particle (RVP) system has identified a novel glycosylation site at residues 270–272 and a specific substitution at residue 272 that reduces neutralization. Site 1 (residues 67 and 83) overlaps the binding footprints of protective monoclonal antibody (mAb) 5A (37), while the exact role of each residue remains undetermined.

In this study, by using reverse genetics, we demonstrated that the substitution of alanine to glutamic acid at position 83 of the E protein is critical for the reduced neutralization sensitivity to the YF-17D-induced antibodies, and recombinant YF-17D carrying the A83E substitution is both safe and immunogenic in mice, representing a promising YF vaccine candidate that deserves further development.

## RESULTS

### Construction and characterization of recombinant YFVs with SAI-specific mutation

**Fig 1:**
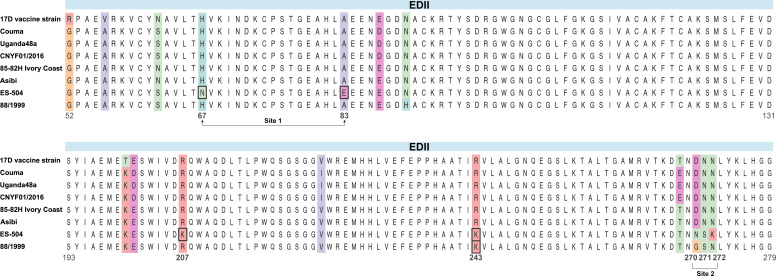
Amino acid polymorphisms of YFVs in EDII. The alignment of amino acid sequences for EDII is presented for the specified sequences. The colored residues within the alignment represent positions where the identity of the residues differs among the eight sequences. Site 1, corresponding to positions 67 and 83, and Site 2, encompassing positions 270–272, are marked below the alignment.

**Fig 2:**
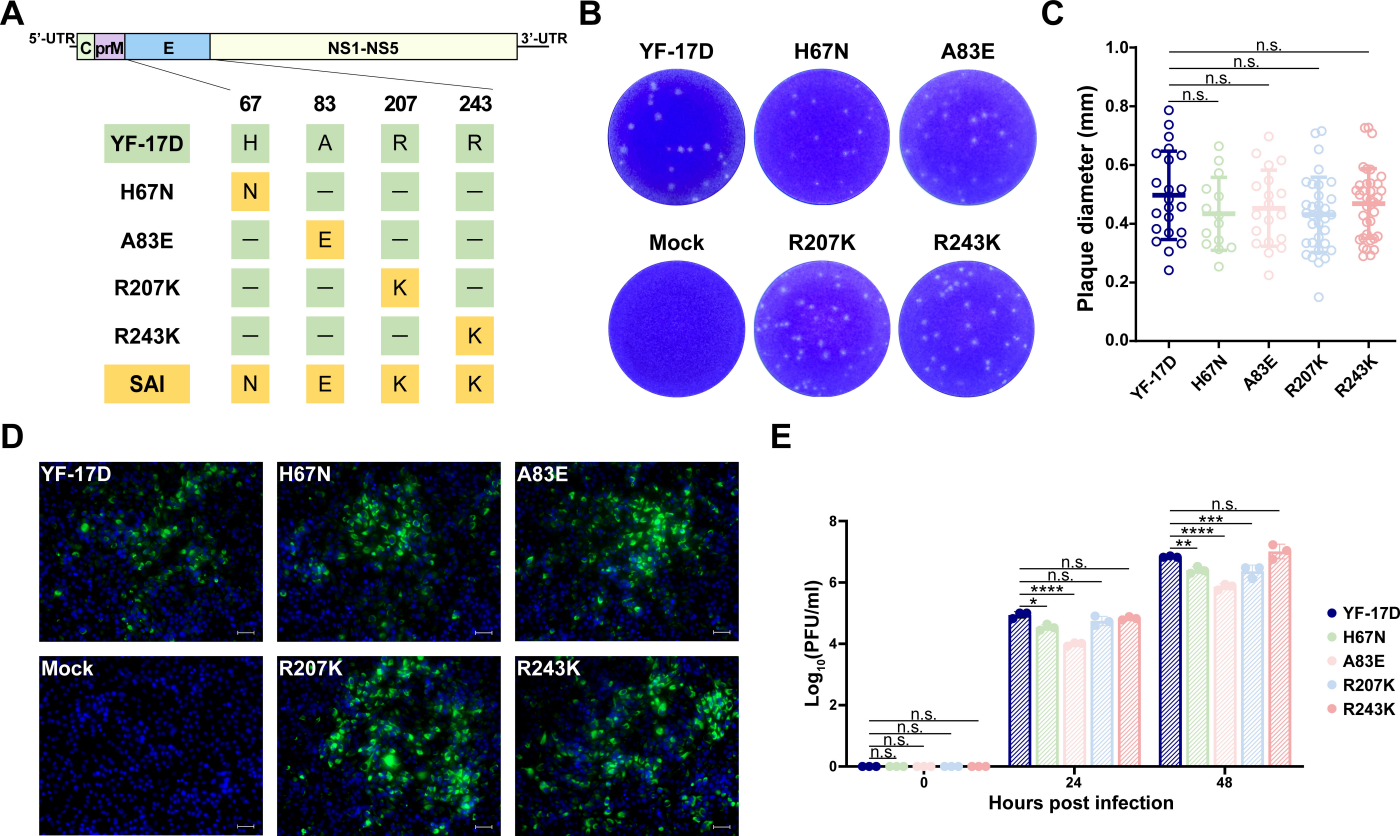
Construction and characterization of mutant YFVs. (**A**) Schematic and mutation sites of YFVs. Unique amino acid residues in EDII are highlighted in green (YF-17D) or yellow (SAI). (**B**) Plaque morphology of YF-17D and mutant YFVs in BHK-21 cells. (**C**) Plaque size of YF-17D and mutant YFVs in BHK-21 cells. YF-17D, *n* = 22; H67N, *n* = 14; A83E, *n* = 19; R207K, *n* = 33; R243K, *n* = 36 (*n*, number of plaques). Statistical significance was determined using Student’s unpaired *t*-test. (**D**) Immunofluorescence staining specific to YFV antigens of mutant YFVs in BHK-21 cells at 48 h post-infection (scale bars = 50 µm). The E protein is stained in green, while the nuclei are stained in blue. (**E**) Growth curves of mutant YFVs in BHK-21 cells. Cells were infected with the indicated viruses at a multiplicity of infection of 0.01. Infectious virus particles in the supernatants at 0, 24, and 48 h post-infection were measured by plaque assays in BHK-21 cells. The data are presented as mean ± SD. Statistical significance was determined using two-way analysis of variance. *P < 0.05, **P < 0.01, ***P < 0.001, ****P < 0.0001. n.s., not significant.

The 2017–2019 YFV epidemics in Brazil, attributed to the SAI YFV strain, prompted us to investigate unique changes in EDII through the alignment of amino acid sequences. Our analysis revealed a total of seven conserved residues unique to SAI YFV (residues 67, 83, 207, 243, 270, 271, and 272) in EDII compared to both YF-17D (GenBank: NC_002031) and its parental strain Asibi (GenBank: KF769016) (Fig. 1). Considering the well-documented role of glycosylation site residues 270–272 (NSK) (21), our subsequent analysis concentrated on the remaining four amino acids substitutions (H67N, A83E, R207K, and R243K). To elucidate the specific impact of these individual mutations, we utilized reverse genetics techniques to recover four mutant YFV strains, each containing a single amino acid substitution, using YF-17D as the genetic backbone (Fig. 2A). As expected, all mutant YFVs demonstrated viability post-transfection and exhibited plaque phenotypes on BHK-21 cells that were comparable to YF-17D (Fig. 2B and C). IFA showed the expression levels of the E protein in BHK-21 cells infected with all mutant viruses were similar to that observed with YF-17D (Fig. 2D). Furthermore, growth curve analysis showed that all mutant viruses were capable of replication in BHK-21 cells (Fig. 2E).

### The A83E mutation reduces neutralization sensitivity to YF-17D-vaccinated sera

**Fig 3:**
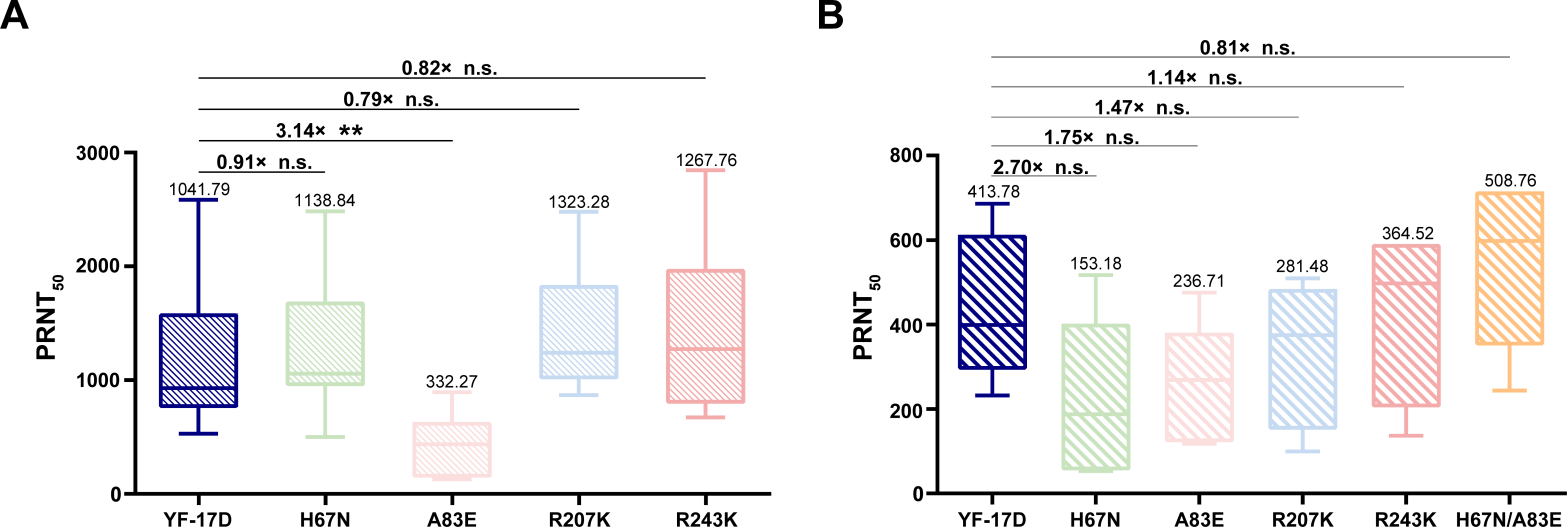
Neutralizing sensitivity of mutant YFVs to YF-17D-vaccinated mouse and human sera. Neutralizing titers of YF-17D-vaccinated mouse (**A**) or human (**B**) sera to mutant viruses were measured using plaque assays in BHK-21 cells. Geometric mean titers and the fold change reduction of neutralizing titers compared to YF-17D are shown above each data set. Statistical significance was determined using Student’s unpaired *t*-test.**P < 0.01. n.s., not significant.

Further, we aimed to assess the susceptibility of each mutant to neutralization by YF-17D-immunized mouse sera. The standard plaque reduction neutralization test (PRNT) demonstrated that the A83E mutant exhibited a notable decrease in sensitivity to neutralization (Fig. 3A), with a geometric mean titer (GMT) that was 3.14-fold lower than that of YF-17D. Conversely, no significant difference was observed between the YF-17D and the other three mutants, including H67N, R207K, and R243K.

Furthermore, we assessed the neutralizing capability of sera from a cohort of YF-17D vaccinees (*n* = 5) against each single mutant and the H67N/A83E mutant mutant. As shown in Fig. 3B, despite no statistical difference being observed, all four mutant viruses carrying a single substitution showed reduced neutralization sensitivity to the vaccinee sera. Notably, the H67N and A83E mutants displayed 2.7- and 1.75-fold reductions in GMT titers, respectively. Interestingly, the H67N/A83E double mutant showed no obvious reduction in neutralization sensitivity to vaccine sera (Fig. 3B). Thus, among the four amino acid substitutions, a single A83E substitution in the EDII conferred reduced neutralizing sensitivity to sera from YF-17D-vaccinated mice and humans.

### The A83E mutant virus maintains the attenuation phenotype in mice

**Fig 4:**
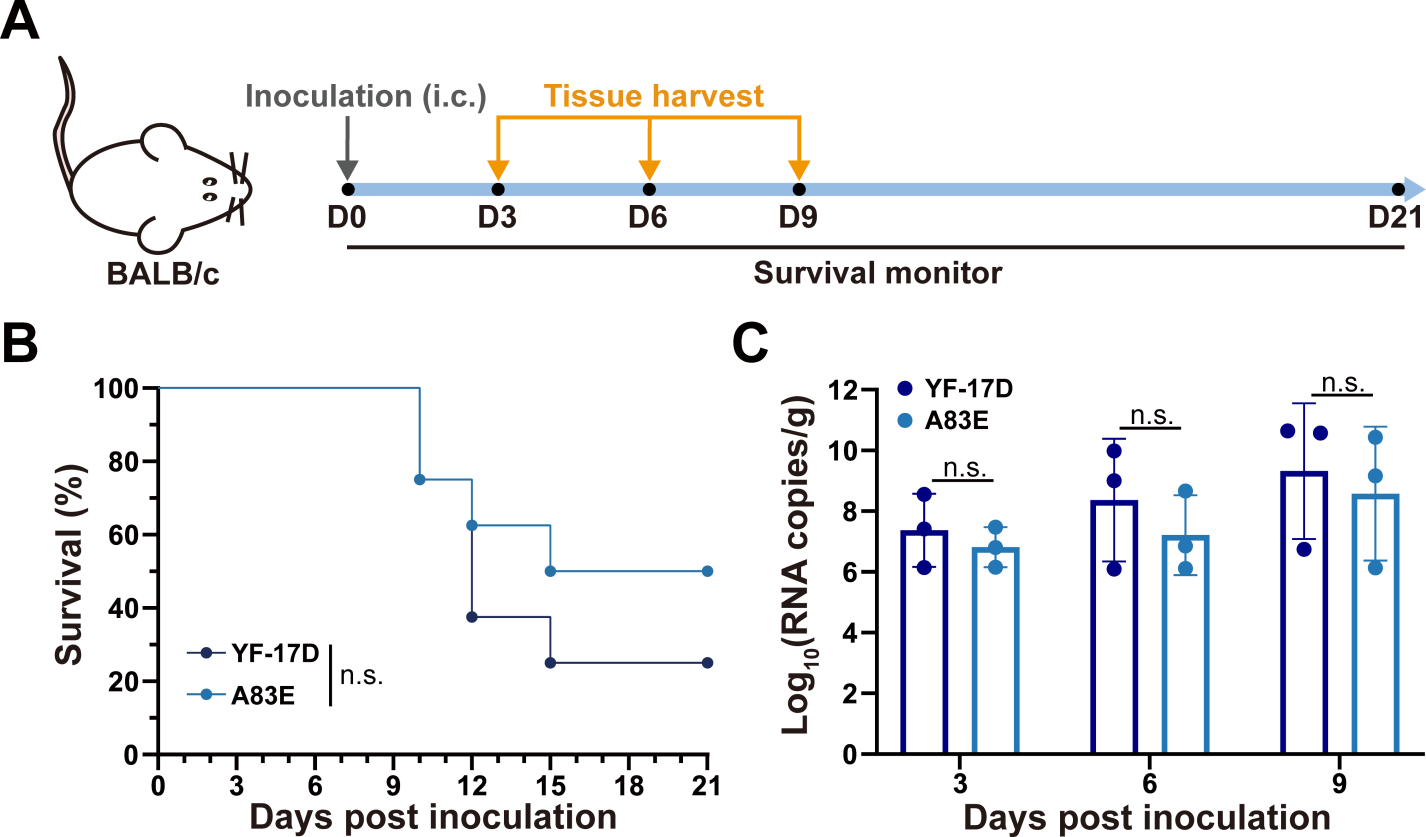
Neurovirulence of the mutant virus A83E in adult mice. (**A**) Experimental workflow overview. Six-week-old female BALB/c mice were intracranially inoculated with 1 PFU of YF-17D or A83E. Survival was monitored for 21 days. Brain samples were collected on 3, 6, and 9 days post-inoculation. (**B**) Survival curve of infected mice (*n* = 8). Statistical significance was determined using a log-rank test. (**C**) Viral loads in brains of infected mice (*n* = 3). Viral RNA in mouse brains was quantified by reverse transcription quantitative PCR. The data are shown as mean ± SD. Statistical significance was determined using two-way analysis of variance. n.s., not significant.

To better understand the biological characteristics of the A83E mutant, we compared the neurovirulence and neuroinvasiveness between the A83 mutant and YF-17D using established mouse models (38–40). The neurovirulence test in adult BALB/c mice (Fig. 4A) showed that both YF-17D and A83E exhibited comparable mortality rates following intracranial injection. In addition, the mean survival time for the A83 group was slightly extended by 2.5 days in comparison to the YF-17D group (15.9 days vs 13.4 days) despite the absence of statistically significant differences (Fig. 4B). The infected mice displayed outward symptoms including dyspnea, tremor, hunched posture, and hind-limb paralysis. Furthermore, an analysis of viral RNA levels in mouse brains showed no significant differences between YF-17D and the A83E mutant (Fig. 4C).

**Fig 5:**
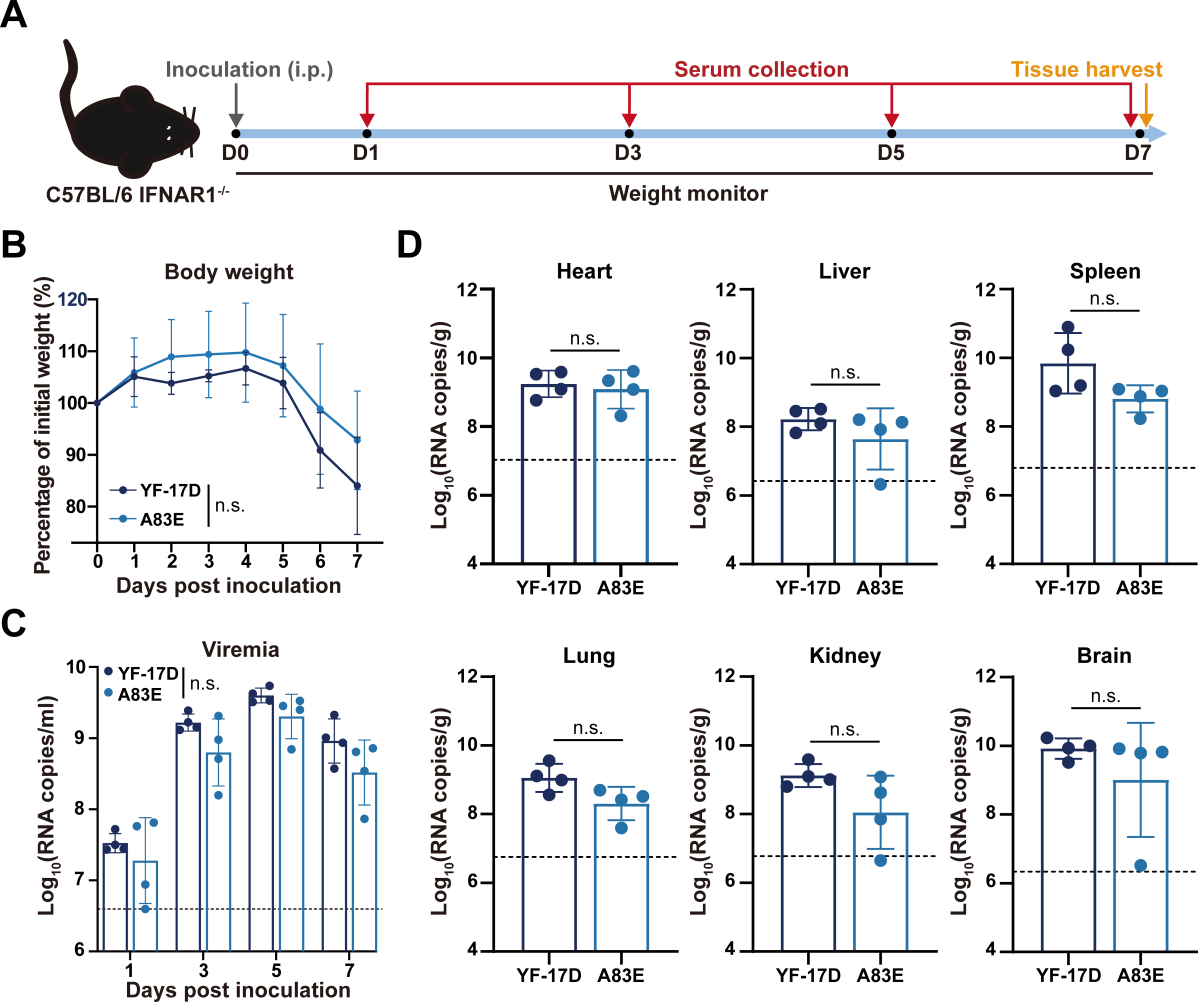
The mutant virus A83E shows neuroinvasiveness similar to YF-17D. (**A**) Experimental workflow overview. Three- to four-week-old female IFNAR1^−/−^ mice (*n* = 4) were intraperitoneally inoculated with 10^5^ PFU of YF-17D or A83E, and daily weight monitoring was performed. Serum samples were collected on 1, 3, 5, and 7 days post-inoculation, and tissues were harvested at 7 days post-inoculation. (**B**) Body weight changes of inoculated mice. (**C**) Viremia was measured using RT-qPCR. (**D**) Assessment of viral loads in various tissues of inoculated mice at 7 days post-infection. In all panels, data are shown as mean ± SD. Groups in panels** B and C** were compared by two-way analysis of variance. Groups in panel** D** were compared using Student’s unpaired *t*-test. n.s., not significant.

We assessed the neuroinvasiveness of YFVs using 3- to 4-week-old IFNAR1^−/−^ C57BL/6 mice as previously described (39). The experimental procedure is illustrated in Fig. 5A. Following intraperitoneal injection, all animals injected with either YF-17D or A83E showed a decrease in body weight starting from 5 days post-inoculation (dpi), with no significant differences observed between the two groups (Fig. 5B). Both YF-17D and A83E challenges led to sustained viremia from 2 to 7 dpi, with similar peak viral RNA loads and kinetics (Fig. 5C). Viral RNAs were detected in all collected tissues, including the heart, liver, spleen, lung, kidney, and brain at 7 dpi, with no significant differences between the two groups (Fig. 5D). In conclusion, these results demonstrate that the A83E mutant virus exhibits a safety profile comparable to YF-17D in mice, suggesting that the A83E mutation in the YFV E protein does not significantly affect its neurovirulence and neuroinvasiveness.

### The A83E mutant virus elicits a robust immune response in mice

**Fig 6:**
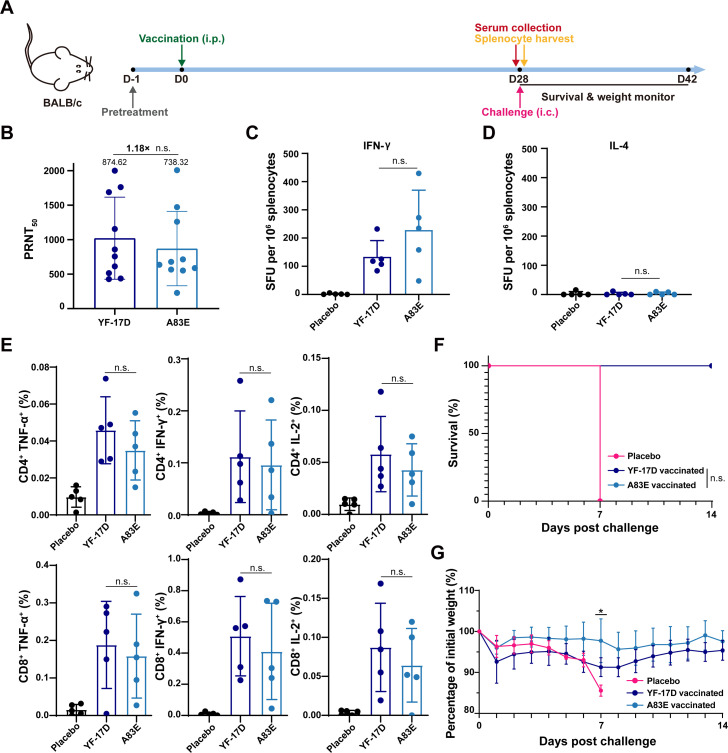
A single vaccination with A83E induces enhanced neutralization and complete protection in mice. (**A**) Flowchart of vaccination and challenge experiment. Six-week-old female BALB/c mice were pretreated with MAR1-5A3 1 day before vaccination. Serum and splenocytes were collected at 28 days post-vaccination. Vaccinated mice were challenged at 28 days post-vaccination. Survival and weight were monitored for 14 days. (**B**) Sera neutralizing titers (PRNT_50_) of YF-17D and A83E for A83E-vaccinated BALB/c mice (*n* = 10) at 28 days post-vaccination. (**C and D**) ELISpot assay for IFN-γ and IL-4 in splenocytes. (**E**) YFV-specific CD4^+^ and CD8^+^ T-cell responses following vaccination were detected by flow cytometry. (**F**) Survival curve of challenged mice (*n* = 6). Vaccinated mice were challenged with 1,000 PFU of YF-17D, and survival was monitored for 14 days. (**G**) Body weight changes of challenged mice. Data are shown as mean ± SD. Groups in panels B–E were compared using Student’s unpaired *t*-test. Groups in panel **F** were compared using the log-rank test. Groups in panel** G** were compared by two-way analysis of variance. *P < 0.05. n.s., not significant; PRNT_50_, 50% plaque reduction neutralization test.

Due to the comparable safety profiles of the A83E mutant virus and the licensed vaccine strain YF-17D in mice, we proceeded to assay their immunogenicity and protection in mice. The experimental design is illustrated in Fig. 6A. As shown in Fig. 6B, a single immunization with A83E resulted in the generation of a high nAb titer against the A83E virus. Importantly, immunization with A83E also induced nAb titers against YF-17D that were comparable to those against A83E (Fig. 6B). The cellular immune response is critical for vaccination-mediated protection against YFV (41, 42). Subsequently, we compared the T-cell response induced by A83E and YF-17D vaccination. Enzyme-linked immunosorbent spot (ELISpot) assay conducted on splenocytes collected at 28 days post-vaccination showed robust secretion of IFN-γ in mice vaccinated with either A83E or YF-17D. In contrast, IL-4 secretion was either absent or near the detection limits in all vaccinated animals (Fig. 6C and D), suggesting a Th1-prone cellular response. In line with this, flow cytometric analysis revealed robust and YFV-specific intracellular cytokine production in CD4^+^ and CD8^+^ T cells from the spleens of vaccinated mice upon stimulation with the YF-17D E peptide (Fig. 6E).

Finally, a lethal challenge model was used to test the protective effect of A83E and YF-17D vaccination. Following an intracranial challenge, the mice in the placebo group succumbed within 7 days post-challenge as expected, while all animals receiving vaccination with either A83E or YF-17D survived without exhibiting any clinical symptoms (Fig. 6F and G). All together, these results clearly show that a single vaccination with A83E not only induced an enhanced neutralization capability compared with YF-17D but also conferred complete protection against lethal YFV challenge.

## DISCUSSION

Here, we conducted the amino acid sequence alignment of EDII to screen a set of conserved amino acid changes between the YF-17D vaccine strain and SAI YFV strains. These identified amino acid changes were individually introduced into the YF-17D genome using reverse genetics to generate a panel of mutant viruses. Through neutralizing activity assays and mouse experiments, we observed that an amino acid change from A to G at position 83 of the E protein significantly reduced the neutralizing sensitivity of YF-17D-vaccinated mouse sera against the corresponding mutant virus. Importantly, vaccination with A83E induced higher levels of neutralizing antibodies against the mutant virus compared to YF-17D, suggesting the critical role of this amino acid residue in defining neutralizing epitopes on EDII.

The primary goal of our study is to identify the critical amino acid residues that contribute to the reduced neutralizing sensitivity to YF-17D-vaccinated sera (21). Based on the RVP system, Haslwanter et al. have demonstrated that amino acid substitutions at site 1 (H67N and A83E) in EDII have minimal influence on YF-17D neutralization by YF-17D vaccinee sera (21). Consistently, our analysis with reverse genetics also showed similar results (Fig. 3B). Interestingly, the A83E mutant showed reduced neutralization sensitivity to YF-17D-vaccinated sera from both mice and humans. A set of nAbs recognize complex quaternary epitopes spanning multiple E subunits in intact viral particles of other flaviviruses (43). Previous studies with YFV and DENV suggest that quaternary epitope binders may account for a significant portion of neutralizing activity in human immune sera (36, 44). The residue at position 83 is located in a sequence near the fusion loop containing or overlapping the epitopes of the protective nAb 5A (35, 37). We propose that the A83E mutation may elicit allosteric alterations in the E protein, which could potentially influence epitope accessibility and interfere with the availability of other epitopes (45–47). Further investigation is needed to fully elucidate the underlying mechanisms involved.

Notably, our neutralization sensitivity results from mouse and human sera are not entirely consistent (Fig. 3), and similar phenomena have been documented previously. For example, EDIII-specific antibodies are prominent in the mouse immune response (48, 49), but other reports have suggested that EDIII responses formed only a tiny proportion of the total antibody response in human patients infected with DENV or WNV (50, 51). Furthermore, the majority of neutralizing activity has been attributed to antibodies targeting alternative sites on the E protein (48, 50, 52, 53). With regard to YFV, recent serum depletion studies have shown that a large fraction of the YFV E-specific serum antibody response is directed toward EDI and/or EDII, with minimal or low levels of antibodies targeting EDIII (36). Moreover, a recent study showed the nAb response to YF-17D is primarily mediated by antibodies recognizing epitopes located near the FL in EDII (36). This observation is consistent with findings on human monoclonal antibodies targeting similar epitopes on EDII (23, 37). Therefore, considering the majority of flavivirus antibodies in humans target regions outside of EDIII, we hypothesize that alterations in the amino acid composition of EDII may have a more pronounced influence on the modulation of the neutralizing activity of human serum. Additionally, due to resource limitation, the cohort used for human sera neutralization analysis in our study is small (*n* = 5). Further analysis with more clinical samples from vaccinated or infected individual would strengthen our present findings.

YFV-specific nAbs are generally considered as a correlate of protection following YF vaccination. In most clinical trials evaluating flavivirus vaccines, including the YF vaccine, a 50% plaque reduction neutralization test (PRNT_50_) with a threshold titer of 1:10 is utilized as a correlate of protection (54–56). More importantly, recent findings by Kareko et al. suggest that 20% of YF-17D vaccinees will lack detectable neutralizing antibodies 10 years post-vaccination (57). Our investigation revealed that the A83E mutant virus exhibited 3.14- and 1.75-fold decreases in serum neutralizing titers, as determined by PRNT_50_, in sera from mice immunized with YF-17D and from human vaccine recipients, respectively. Consequently, our observations raise concerns regarding an increased risk for individuals immunized with YF-17D during outbreaks of emerging YFV strains harboring the A83E mutation. The gold-standard non-human primate model effectively mirrors key aspects of immune response to vaccination and disease progression. Nonetheless, mouse models exhibiting clinical and pathologic changes similar to human cases have also been developed. For example, IFNAR1^−/−^ mice serve as a sensitive model for the examining the viscerotropism and neurotropism of YFV after subcutaneous injection, which can help evaluate vaccine candidate attenuation properties in accordance with established safety criteria (38, 39). The A83E mutant virus displayed phenotypes in neurovirulence and viscerotropism that are comparable to those of YF-17D in BALB/c and IFNAR1^−/−^ C57BL/6 mice, respectively (Fig. 4 and 5). This observation underscores the potential of the conserved SAI-specific residue A83 as a target for the update of the current gold-standard vaccine.

Taken together, our study identifies a key amino acid at position 83 of the YF-17D E protein in determining neutralizing epitopes of the virus while maintaining the safety profile associated with the YF-17D vaccine. This amino acid residue may serve as a potential target for developing a new generation of live-attenuated YFV vaccines.

## MATERIALS AND METHODS

### Cells and viruses

Baby hamster kidney fibroblast cell line BHK-21 (ATCC CCL-10) were maintained in Dulbecco’s Modified Eagle Medium (DMEM, high glucose; Gibco) supplemented with 10% fetal bovine serum (FBS, Gibco), 1% Penicillin/Streptomycin (Gibco), and 50 mM HEPES (Gibco) in an incubator at 37°C with 5% CO_2_. The YF-17D vaccine strain was recovered from the infectious clone pACNR-FLYF17Dx. Mutant YFVs were recovered from infectious clones containing indicated mutation sites based on the pACNR-FLYF17Dx. Viral stocks were produced in BHK-21 cells, titrated using plaque assays with BHK-21 cells, aliquoted, and then stored at −80°C. All virus experiments were conducted using biosafety level 2 protocols approved by the Institutional Biosafety Committee at the Beijing Institute of Microbiology and Epidemiology.

### Sera from YF-17D vaccinees

Volunteers with history of YFV vaccination who consented to participate were recalled for plasma collection at time points ranging from 5 to 9 years after the first YFV vaccination. Collected plasmas were stored frozen in aliquots at −80°C.

### Alignment of YFV amino acid sequences

Amino acid sequence alignment of EDII was performed using MEGA 11 software (version 11.0.13). These sequences were retrieved from the National Center for Biotechnology Information (Table 1) and aligned with the wild-type YFV strain Asibi and the live-attenuated vaccine strain YF-17D. The alignment results were visualized using the R software.

**TABLE 1:** Origin of YFVs analyzed in sequence alignment

Accession no.	Strain	Origin	Year	Genotype
NC_002031	17D vaccine strain	N/A	N/A	N/A
DQ235229	Couma	Ethiopia	1961	East/Central Africa
AY968065	Uganda48a	Uganda	1948	East Africa
KU921608	CNYF01/2016	China	2016	Angola
U54798	85-82H Ivory Coast	Ivory Coast	1982	West Africa I
KF769016	Asibi	Ghana	1927	West Africa II
KY885000	ES-504	Brazil	2017	South America I
KF907504	88/1999	Bolivia	1999	South America II

### Generation of mutant YFVs

The Q5 site-directed mutagenesis kit (NEB) was utilized to introduce amino acid substitutions (H67N, A83E, R207K, R243K, and H67N/A83E) into the infectious clone pACNR-FLYF17Dx. The infectious clone plasmids were linearized using restriction endonuclease digestion and purified with phenol/chloroform extraction. Viral RNA was produced by *in vitro* transcription with RiboMAX large-scale RNA production system-SP6 (Promega) and purified using PureLink RNA mini kit (Thermo Fisher Scientific). The RNA transcripts were then transfected into BHK-21 cells using Lipofectamine 3000 reagent (Thermo Fisher Scientific). Culture supernatants were harvested upon observation of typical cytopathic effects and stored at −80°C for further use. The titers of virus stocks were determined by plaque assay, and the substitution sites were confirmed by sequencing.

### Plaque assay

Virus samples were 10-fold diluted with DMEM supplemented with 2% FBS, and 300 µL of each dilution was added to BHK-21 cell monolayers in 12-well plates followed by a 1 h incubation at 37°C with 5% CO_2_. The supernatants were discarded, and the cell monolayers were washed twice with phosphate-buffered saline (PBS). Then, each well was overlaid with a mixture of 1 mL DMEM containing 2% FBS and 1% low-melting point agarose (Promega). The infected cells were then incubated at 37°C with 5% CO_2_ for 3–4 days and then fixed with 4% paraformaldehyde (Biosharp), followed by staining with 1% crystal violet solution. Plaques were counted for the calculation of virus titers.

### Immunofluorescence assay

Infected BHK-21 cells were fixed with a cold mixture of methanol/acetone (vol/vol, 7/3) for 30 min. Then, coverslips were incubated with the YFV E protein antibody (GeneTex, GTX134024) at 37°C for 1 h, followed by three washes with PBS. The coverslips were then incubated with CoraLite 488-conjugated goat anti-rabbit IgG antibody (Proteintech, SA00013-2) for 1 h at 37°C and washed as described above. The nucleus was stained with 4′,6-diamidino-2-phenylindole (Invitrogen), and fluorescent cells were examined and imaged using a fluorescence microscope (Zeiss, Germany).

### Growth curves

Growth curves of viruses were assessed by infecting BHK-21 cells at a multiplicity of infection of 0.01. The viral supernatants were replaced with DMEM supplemented with 2% FBS after 1 h of incubation. Infected cells were maintained at 37°C with 5% CO_2_, and supernatants were collected at the indicated time points. Viral titers were quantitated by plaque assay on BHK-21 cells or by RT-qPCR.

### PRNT

The neutralizing antibody titers of sera were determined by a PRNT_50_. Threefold serial dilutions of human or mouse sera were inactivated at 56°C for 30 min and incubated for 1 h at 37°C with an equal volume of diluted virus solution containing about 100 PFU of YFVs or the corresponding mutants. After neutralization, the mixture was added to BHK-21 cell monolayers in 12-well plates, followed by a 1 h incubation at 37°C with 5% CO_2_. The following steps were the same as the plaque assay described above. The PRNT_50_ titers were calculated using the method of Spearman-Karber.

### RT-qPCR for RNA quantification

Viral RNA was extracted using the QIAamp Viral RNA Mini Kit (QIAGEN) according to the manufacturer’s instructions. RNA quantification was performed by targeting the NS5 gene of YFV using One-Step PrimeScript RT-PCR Kit (Takara, RR064A) with the following primers and probe: YFV-F (5′-GCACGGATGTGACAGACTGAAGA-3′), YFV-R (5′-CCAGGCCGAACCTGTCAT-3′), and YFV probe (5′-CGACTGTGTGGTCCGGCCCATC-3′). Standard curves were generated using a 10-fold serial dilution of *in vitro* transcribed YFV viral RNA to quantify copies of the YFV genome.

### Mouse neurovirulence and neuroinvasiveness tests

BALB/c mice were used for experiments involving neurovirulence and vaccination. C57BL/6 mice deficient in the IFN-α/β receptor (IFNAR1^−/−^) were used for neuroinvasiveness tests. For the neurovirulence test, 6-week-old female BALB/c mice were intracranially inoculated with 1 PFU of YFVs. The survival of the mice was monitored daily for 21 days following inoculation. In the neuroinvasiveness experiment, 3- to 4-week-old IFNAR1^−/−^ mice were intraperitoneally infected with 10^5^ PFU of indicated viruses or PBS. The mice were monitored for weight change for 7 days. Tissues were collected for viral load detection using RT-qPCR.

### Mouse vaccination and challenge experiments

For the immunity test, 6-week-old female BALB/c mice were pretreated with the anti-mouse IFNAR1 mAb MAR1-5A3 (IgG1; Leinco Technologies, St. Louis, MO, USA) 1 day before vaccination. Then, mice were intraperitoneally vaccinated with 2 × 10^4^ PFU of YFVs. Serum and spleen samples were collected at 28 days post-vaccination. Vaccinated mice were challenged intracranially with 10^3^ PFU of YF-17D at 28 days post-vaccination. Survival and weight loss were monitored for 14 days.

### ELISpot assay

Cellular immune responses in the vaccinated mice were assessed using IFN-γ and IL-4 precoated ELISpot kits (MabTech). Briefly, the plates were blocked using RPMI 1640 (Thermo Fisher Scientific) containing 10% FBS and incubated for 30 min. Splenocytes were plated at 96-well plates (3 × 10^5^ cells/well) using a pool of overlapping 15-amino acid peptides covering the E protein of YF-17D. Concanavalin A (Sigma) and RPMI 1640 media were used as positive and negative controls, respectively. After incubation at 37°C with 5% CO_2_ for 36 h, biotinylated anti-mouse IFN-γ and IL-4 antibodies were added to each well, followed by incubation for 2 h at room temperature. After adding the AEC substrate solution, the air-dried plates were read using the automated ELISpot reader (AID). The numbers of spot-forming cells (SFCs) per 10^6^ cells were calculated. The medium background levels were typically <3 SFC per 10^6^ cells.

### Flow cytometry analysis for mouse splenocytes

T-cell proliferation in vaccinated mice was evaluated using a FACSCalibur flow cytometer (BD Biosciences). Briefly, 1 × 10^6^ mouse splenocytes were stimulated with a YF-17D peptide pool for 2 h at 37°C with 5% CO_2_. Brefeldin A (1 mg/mL, MCE) was then added into splenocytes and incubated for 4 h. The splenocytes were permeabilized and stained with fluorescently conjugated antibodies to CD3 (PE/Cyanine7) (BioLegend), CD4 (FITC) (BioLegend), and CD8 (APC/FITC) (BioLegend). Dead cells were stained with a Zombie UV3 fixable viability Kit (BioLegend). Data were analyzed with FlowJo software.

### Statistical analysis

Data analysis was carried out using the GraphPad Prism software. Log-rank tests were performed for the survival analysis. Two-way analysis of variance was utilized for double-factor analysis, while Student’s unpaired *t*-test was employed for single-factor analysis. *P* values are denoted as follows: n.s. or not significant, *P < 0.05, **P < 0.01, ***P < 0.001, ****P < 0.0001.

## Data Availability

The data associated with this paper are available upon request to the corresponding author.
